# In Vitro Evaluation of Glycoengineered RSV-F in the Human Artificial Lymph Node Reactor

**DOI:** 10.3390/bioengineering4030070

**Published:** 2017-08-15

**Authors:** Lars Radke, Grit Sandig, Annika Lubitz, Ulrike Schließer, Hans Henning von Horsten, Veronique Blanchard, Karolin Keil, Volker Sandig, Christoph Giese, Michael Hummel, Stephan Hinderlich, Marcus Frohme

**Affiliations:** 1 Molecular Biotechnology and Functional Genomics, Technical University of Applied Sciences Wildau, Hochschulring 1, Wildau 15745, Germany; lars.radke@charite.de (L.R.); keil@th-wildau.de (K.K.); 2 Institute of Pathology, Charitè-University Medicine Berlin, Augustenburger Platz 1, Berlin 13353, Germany; michael.hummel@charite.de; 3 Laboratory of Biochemistry, Department of Life Sciences and Technology, Beuth University of Applied Sciences, Seestraße 64, Berlin 13347, Germany; Grit.Sandig@HTW-Berlin.de (G.S.); hinderlich@beuth-hochschule.de (S.H.); 4 ProBioGen AG, Goethestraße 54, Berlin 13086, Germany; Annika.Lubitz@Probiogen.de (A.L.); Ulrike.Schliesser@Probiogen.de (U.S.); Volker.Sandig@Probiogen.de (V.S.); Christoph.Giese@Probiogen.de (C.G.); 5 Department of Life Science Engineering, HTW Berlin University of Applied Sciences, Wilhelminenhofstraße 75a, Berlin 12459, Germany; HansHenning.vonHorsten@htw-berlin.de; 6 Institute of Laboratory Medicine, Clinical Chemistry and Pathobiochemistry, Charité Medical University Berlin, Augustenburger Platz 1, Berlin 13353, Germany; veronique.blanchard@charite.de

**Keywords:** Glycoengineering, fucosylation, RSV, F-Protein, NanoString

## Abstract

Subunit vaccines often require adjuvants to elicit sustained immune activity. Here, a method is described to evaluate the efficacy of single vaccine candidates in the preclinical stage based on cytokine and gene expression analysis. As a model, the recombinant human respiratory syncytial virus (RSV) fusion protein (RSV-F) was produced in CHO cells. For comparison, wild-type and glycoengineered, afucosylated RSV-F were established. Both glycoprotein vaccines were tested in a commercial Human Artificial Lymph Node in vitro model (HuALN^®^). The analysis of six key cytokines in cell culture supernatants showed well-balanced immune responses for the afucosylated RSV-F, while immune response of wild-type RSV-F was more Th1 accentuated. In particular, stronger and specific secretion of interleukin-4 after each round of re-stimulation underlined higher potency and efficacy of the afucosylated vaccine candidate. Comprehensive gene expression analysis by nCounter gene expression assay confirmed the stronger onset of the immunologic reaction in stimulation experiments with the afucosylated vaccine in comparison to wild-type RSV-F and particularly revealed prominent activation of Th17 related genes, innate immunity, and comprehensive activation of humoral immunity. We, therefore, show that our method is suited to distinguish the potency of two vaccine candidates with minor structural differences.

## 1. Introduction

In recent years, the process of vaccine development has evolved drastically. Yet, the main requirement on vaccines have not changed: the establishment of a long-lasting and pathogen-specific immunological memory as a result of a concerted immune response. Subunit vaccines are highly focused and specific, but often suffer from reduced immunogenicity, low reactogenicity, and limited availability of innate defense triggers [1]. Although adjuvants are applied to compensate for these deficiencies by activation of different cell types or by a long-lasting release of antigen, the frequently-used aluminum salts and mineral oil lead to diminished immune responses or side effects [2]. Recently, in vitro experiments showed that the adjuvant component α tocopherol elevated the risk of narcolepsy after H1N1 vaccination [3]. Covalent linkage of immunogenic agents with the vaccines, e.g., by cross-linking, could be used for the enhancement of efficacy. Recombinant protein vaccines opened a way to introduce immunogenic agents immediately during the production process. This could be done by additional immunogenic peptides, which are bound N- or C-terminally to the protein backbone, or by introduction of non-human posttranslational modifications, e.g., via glycosylation in the case of glycoprotein vaccines [4]. These foreign glycosidic structures are able to interact with the conserved Toll-like receptors and, by that, stimulate the immune system comprehensively. Therefore, they can be used as adjuvants in vaccine development which mainly boost the recognition of very small inoculants, like toxins, subcellular components, or viral proteins. In this study a subunit vaccine candidate against the respiratory syncytial virus (RSV) was used as a model. It was recombinantly produced in its native and afucosylated forms. However, glycans with and without fucose are both common in humans and, therefore, a direct adjuvant role is highly unlikely. This is in contrast to monosaccharides artificial to humans, e.g., N-glycan-bound xylose, which may work as adjuvants [4,5,6].

RSV causes infections of the lower respiratory tracts and has a prevalence of nearly 100% within the first 24 months of life [7]. The virus is a certain threat to prematurely-born children and immunocompromised people. According to estimates, 600,000 people die as a result of RSV infections each year [8]. The common antibody treatment with Palivizumab is of short protective effect, cost intensive, and restricted to infants of high risk. DeVincenzo et al. developed an oral RSV entry inhibitor (named GS-5806) which lowered the viral load and the disease symptoms in a double-blind placebo-controlled exposition study [9]. However, until now, there has been no vaccine available and, recently, a phase III study by Novavax failed to confirm vaccine efficacy [10].

The RSV envelope consists of two major proteins, RSV-G (where G stands for glycoprotein) and the “fusion” protein RSV-F. Both proteins have been used as targets in previous attempts (e.g., [11,12]). In this study, we focused on the highly-conserved RSV-F which is responsible for virus spreading by fusing with neighboring healthy cells. It contains three N-glycans playing a critical role in this process [13]. For vaccine production, a soluble variant of RSV-F was constructed lacking the transmembrane region, but fused with a C-terminal Factor Xa cleavage site and a 6xHis-tag. The construct contains two furin cleavage sites that allow proteolytic processing of RSV-F within the trans-Golgi. This mechanism results in the native active form (disulfide-linked F1 and F2 subunits) and release of the p27 peptide.

It became obvious in recent years that the predictive value of substance testing in animals is limited and the phylogenetic distance between laboratory animals and humans is considerable [14]. RSV vaccines are often tested in mice or cotton rats which, in part, mimic human responses. However, there is no single globally-accepted animal-model for the study of RSV vaccines and none in which RSV disease fully matches that of humans [15]. Several studies failed to transfer positive results from rodents, or even primates [16], to humans. In addition, animal studies are outside the scope of this (preliminary) study. In vitro models exist that, e.g., mimic the pediatric bronchial epithelum via a 3D air-liquid interface model [17] or investigate the growth of RSV in primary cells from the human respiratory tract [18]. However, these models cannot display the well-concerted interaction of immune cells. Therefore, we determined the immunogenic potential of the produced vaccine candidates with the altered glycosylation pattern by performing stimulation experiments in a highly-specialized perfusion reactor system named the Human Artificial Lymph Node reactor (HuALN^®^, ProBioGen AG, Berlin, Germany) [19]. It has been developed to model the human immune system in vitro for testing of biopharmaceuticals and vaccines, assessing immunomodulation, immunogenicity, and immunotoxicity.

The reactor allows long-term co-cultivation of primary human peripheral blood mononuclear cells (PBMCs) and matured dendritic cells (mDC). In a 3D hydrogel matrix cells can migrate to form immune-competent micro-organoid structures and dendritic cells form a network in which B- and T-cells can swarm and cluster. The HuALN^®^ enables the analysis of cellular and humoral immunity on the basis of various read-out parameters, e.g., cytokine profiles, histological studies and the cells can be harvested for further analysis, like gene expression profile studies [20].

Here, we performed standard HuALN^®^ 28-day cultivation experiments, where cells of healthy donors were repeatedly stimulated with mDC and RSV-F variants and cell culture supernatants were collected on a daily basis. The concentrations of six key cytokines were quantified with a bead-based suspension assay (BioPlex, BioRad). These key cytokines were identified in previous studies [21] and cover cellular (IL-2) and humoral (IL-4) immune response, as well as pro- (IFN-g, TNF-a) and anti-inflammatory (IL-10) cytokines and growth factors (GM-CSF). Since cells can only be harvested at the end of the trial, the same experiment was conducted in a fed batch microtiter plate with a comparable stimulation pattern. For a comprehensive gene-expression analysis mRNA was analyzed with a Nanostring nCounter^®^ Gene Expression panel covering more than 500 immunology genes. The most stable expressed reference genes in this experimental set-up were identified in an extensive study beforehand and have been cross-checked with quantitative polymerase chain reaction (qPCR) results [22].

## 2. Materials and Methods

### 2.1. Production and Analysis of Recombinant RSV-F Protein

Cells for expression of soluble RSV-F were obtained according to Sandig et al. [5]. In brief, a cDNA was constructed containing the N-terminal extracellular part of RSV-F, an N-terminal Mellitin signal sequence, and a C-terminal Factor Xa cleavage site fused with a 6xHis-tag. CHO DG44 cells were transfected with the respective vector and selected by puromycin methotrexate. For protein production CHO RSV-F cells were cultivated for 14 days, and RSV F was purified by Ni-NTA affinity chromatography. To obtain glycoengineered RSV-F the cDNA for RMD (GDP-6-deoxy-d-lyxo-4-hexulose reductase) from *Pseudomonas aeruginosa* was co-expressed in CHO RSV-F cells using the GlymaxX^®^ technology [23], resulting in knock-down of the endogenous fucosylation pathway of CHO DG44 cells. In the following we, therefore, indicate RSV-F from CHO DG44 cells as “fucosylated RSV-F” and RSV-F from CHO DG44/RMD cells as “afucosylated RSV-F” or “RSV-F Fuc-”.

The integrity of RSV-F proteins was verified by Western blot analysis using a Penta-His HRP antibody (1:2000, Qiagen, Hilden, Germany). The monosaccharide composition of the proteins was analyzed using high-performance anion exchange chromatography with pulsed amperiometric detection (HPAEC-PAD), as described in [24], and N-glycan patterns by matrix-assisted laser-desorption/ionization time-of-flight (MALDI-TOF) mass spectrometry, as described before [25,26].

### 2.2. Stimulation Experiments

In the cell culture stimulation experiment CD14 (−) PBMC were co-cultured with mDC. Therefore, CD14 (+) and CD14 (−) cells were enriched from whole blood of healthy donors, as described before [21]. Immature dendritic cells (iDC) were differentiated from enriched CD14 (+) cells by the addition of interleukin 4 (IL-4) and granulocyte-macrophage colony-stimulating factor (GM-CSF), 800 U/mL each (both Miltenyi Biotec, Bergisch Gladbach, Germany), on day 1 and 2. Antigen-specific maturation was done on day 7 (1 µg/mL of each antigen). On day 8 all supplements were removed by a media exchange.

Stimulation experiments were conducted simultaneously in three HuALN^®^ reactors to collect and analyze supernatants on a daily basis, as well as in 96-well microtiter plates to harvest cells for a comparative gene expression study of the initial immune response. In two HuALN^®^ reactors CD14 (−) cells were cultivated with mDC and the respective vaccine (fucosylated wild-type RSV-F from parental CHO or afucosylated RSV-F from CHO-RMD). A third HuALN^®^ reactor served as a negative control and was left untreated (no vaccine-specific mDC, and the bolus is media at the days of stimulation).

Cryopreserved PBMCs were thawed and rested for one day at 37 °C, 5% CO_2_. On the next day cells were harvested and adjusted to the following concentrations: each matrix (5 mg/mL agarose and 1.3 mg/mL collagen final concentration) with 500 µL total volume contained 1.7 × 10^7^ DCs (with or without stimulation) and 2 × 10^8^ CD14 (−) cells. Matrices were placed in the HuALN^®^ reactors and perfused with 45.15 µL/h in three intervals of 15 µL/min.

One hundred microliters of supernatants from HuALN^®^ reactors were taken on a daily basis for 28 days (with the exception of days 4, 12, 19, and 25). (Re-) stimulation took place always after collection of supernatants on days 0, 7, 14, and 21.

For the gene expression study 3 × 10^6^/mL CD14 (−) cells with autologous mDCs were seeded in 96-well plates in 200 µL of RPMI 1640 medium supplemented with 10% fetal calf serum. RSV-F variants (1 μg/mL), as well as LPS (10 μg/mL) as a positive control, were added immediately to the cultures. Due to the high cell density, the medium needed to be partially replaced every day as follows: after 24 h, 100 µL cell culture supernatant was taken and 200 µL of new media with stimulants was added to a final volume of 300 µL. Every following media replacement included the collection of 200 µL of supernatant and the addition of 200 µL new media also including stimulants. On day 5 the cells were restimulated with mDC, additionally. After 48 h, five-day and seven-day cells from each treatment were collected by harvesting all cells of a respective well.

### 2.3. Cytokine Analysis

Supernatants from HuALN^®^ reactors were collected over a period of 28 days. Since (re-) stimulation took place after the collection of supernatants, changes in cytokine secretion can be detected one day later, at the earliest. Supernatants were analyzed with a multiplexed suspension array system (Bio-Plex 200, Bio-Rad, Munich, Germany). A Bio-Plex Express assay (Bio-Rad) for six custom analytes was used to quantify the cytokines IL-2, IL-4, IL-10, GM-CSF, IFN-γ, and TNF-α. The assay was performed according to the manufacturer’s protocol, but conducted fully-automated on a Tecan Freedom Evo 200 (Tecan, Männedorf, Switzerland) with an in-house-developed Tecan Freedom EVOware^®^ script in order to lower the variance from manual handling [27]. All samples were tested in duplicate. The results were analyzed with Bio-Plex Manager 6.1 (Bio-Rad, Munich, Germany) using the logistic five-point regression method.

### 2.4. RNA Preparation and NanoString Measurement

Cells from microtiter plates were stored as cell pellets at −80 °C. Extraction of total RNA was performed with a High Pure RNA Isolation Kit (Roche, Mannheim, Germany). The purity of the total RNA was verified with a Nanodrop ND-1000 (Nanodrop Instruments, Wilmington, DE, USA) by determining the spectral absorption quotients 260/280 and 260/230. RNA integrity was checked with a DNF-472 High-Sensitivity RNA Analysis Kit on a Fragment Analyzer™ (Advanced Analytical Technologies, Heidelberg, Germany). Analysis was performed as described by the manufacturer with the exception of an extended runtime of an additional 20 min to be able to detect any remaining contamination with genomic DNA.

RNA samples of vaccine stimulation experiments were analyzed with an nCounter^®^ Gene Expression assay (NanoString^®^ Technologies, Seattle, WA, USA) in duplicate. The prebuilt Human Immunology v2 panel analyzes 579 immunology-related human genes and 15 reference genes in parallel in a special cartridge. RNA samples were quantified with the Qubit^®^ RNA HS AssayKit (Molecular Probes, Eugene, Oregon, USA) immediately prior to preparation for the NanoString experiment. Due to a slight fragmentation of some RNA samples, 150 ng of total RNA was used. NanoString experiments were performed on an nCounter^®^ Dx Analysis System with FLEX Configuration, as described [22].

Data was normalized using nSolver Analysis Software 3.0 (NanoString^®^ Technologies). To estimate the general difference between stimulation types, normalized and log transformed counts of all samples (including the negative control) were utilized for a cluster analysis using hclust in R (version 3.3.2).

Duplicates were grouped and fold-change estimates were calculated within the nSolver software. To test the significance of differentially-expressed genes between both RSV-F variants, the recently-published R package NanoStringDiff [28] was used, which is specially-developed for nCounter assays, where t-test-based approaches do not fit. Herein, counts are normalized, as in the nSolver Analysis Software, and transferred into a generalized linear model of the negative binominal family. An empirical Bayes shrinkage approach is utilized to estimate the dispersion parameters in the used model and differentially-expressed genes are identified using a likelihood ratio test.

Finally, genes were clustered by their function when they were of primary immunologic interest or when they were identified as significantly differentially expressed.

## 3. Results and Discussion

### 3.1. Production and Analysis of RSV-F Proteins

**Figure 1:**
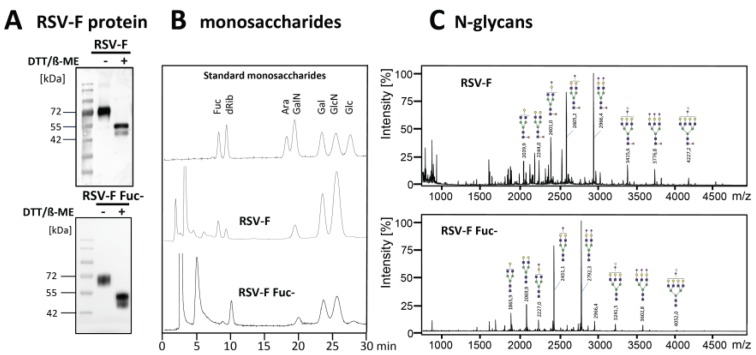
Soluble RSV-F was stably expressed in CHO DG44 cells, as well as in fucose-deficient CHO DG44 cells. RSV-F proteins were purified from cell culture supernatants and analyzed by Western blot. (**A**) Fucose levels of purified and acid-hydrolyzed proteins (15 µg) were analyzed by HPAEC-PAD. (**B**) Peaks were compared to standard monosaccharides: 2-deoxy-D-ribose (dRib), L-fucose (Fuc), D-arabinose (Ara), D-galactosamine (GalN), D-galactose (Gal), D-glucosamine (GlcN), and D-glucose (Glc). N-glycans of the RSV-F proteins were permethylated and masses were obtained by MALDI-TOF mass spectrometry. (**C**) Structures were verified using Glycoworkbench 2.0 [30].

Two glycosylation variants of soluble RSV-F were recombinantly expressed in CHO DG44 and CHO DG44/RMD cells. Proteins were purified from cell culture supernatants by Ni-NTA affinity chromatography. Western blot analysis of the purified proteins displayed proteins of a size of 70 kDa under non-reducing conditions (Figure 1A). Obviously, there are two forms of the RSV-F2 subunit visible under the reducing conditions. We cannot completely rule out that the double band indicates different glycosylation variants [29]. However, it is more likely that differently-processed proteins exist. Although both furin cleavage sites are obviously recognized by the protease, part of RSV-F might only be processed by one furin cleavage and still contain the pep27 peptide, whereas the lower band seems to represent the fully-processed F2 subunit after double furin cleavage. Densitometric estimation of RSV-F and RSV-F Fuc- revealed a ratio of about 70:30 of the upper band (singly-cleaved protein) and the lower band (doubly-cleaved protein) for both samples. The production conditions of RSV-F, therefore, seem to hamper complete posttranslational modification by furin, most likely due to the high expression rate of the recombinant protein and its limited retention time in the Golgi. However, there is no significant effect of fucosylated or afucosylated glycans on furin cleavage efficacy.

Reduction of the fucose level of the RSV-F protein from CHO DG44/RMD cells was proven by monosaccharide analysis via HPAEC-PAD (Figure 1B). The N-glycan patterns of both RSV-F variants were analyzed by MALDI-TOF mass spectrometry. Figure 1C predominantly shows fucosylated N-glycans of RSV-F from CHO DG44 cells. In contrast, analysis of RSV-F from CHO DG44/RMD cells reveals a pattern of comparable N-glycans, but without fucose. These data indicate that RSV-F could be produced successfully as a glycoengineered, non-fucosylated variant in CHO DG 44/RMD cells.

### 3.2. Cytokine Analysis

**Figure 2:**
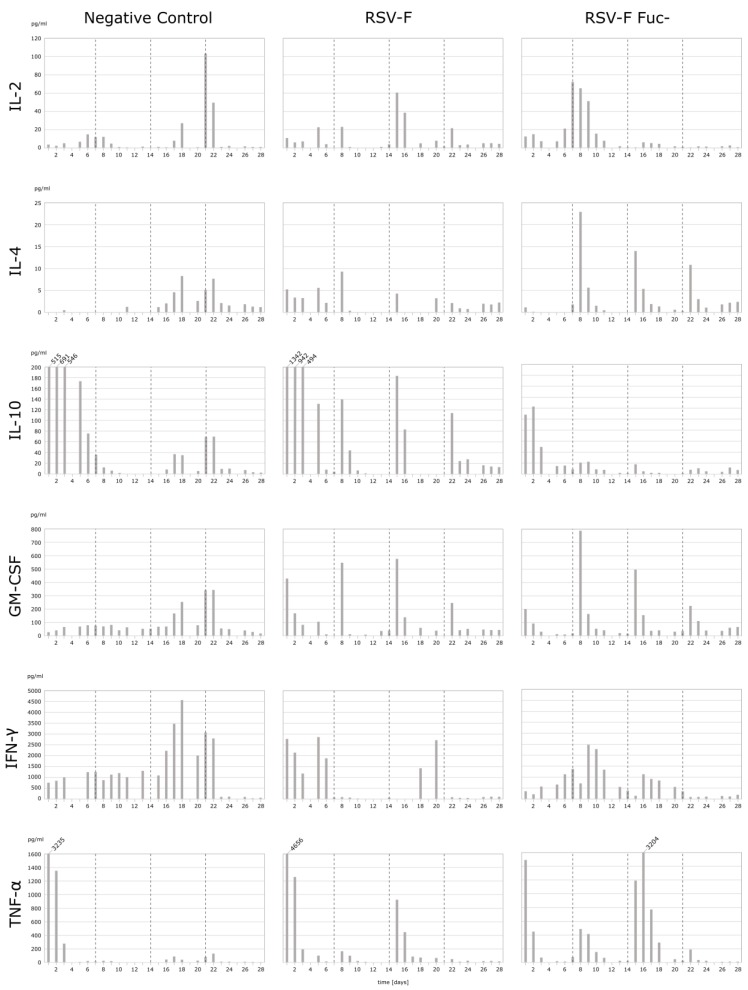
Cytokine secretion (in pg/mL) of PBMC in HuALN^®^ reactors with repeated stimulations. PBMC were incubated for 28 days and stimulated with RSV-F protein variants and the according maturated DC at days 0, 7, 14, and 28 (dotted lines). Some maximum values are cut off (IL-10 and TNF-α) for better resolution of lower concentrations. Since both cytokines can act in an autocrine-like manner, the height of the values is of minor importance, immunologically, if a certain threshold is exceeded.

Three HuALN^®^ reactors were run with fucosylated RSV-F, RSV-F Fuc-, and one negative control. Quantification of the secreted cytokines in the cell culture supernatants was used to evaluate the extent of the humoral and cellular activation of the immune cells. Since only 100 µL of supernatants were available per day, multiplexed assays were required to quantify the six key cytokines IL-2, IL 4, IL-10, GM-CSF, IFN-γ, and TNF-α. Bead-based suspension assays allow the quantification of up to 100 analytes in as little as 50 µL, thus enabling the measurement of duplicates. Bio-Plex Express Kits were used with the help of an automated liquid handling robot in order to achieve very high precision and repeatability. Mean recovery rates of spike-in-controls were between 91% and 122%, and for 70% of all data points error limits were below 20% (median error rate 10.4%). The time course of the cytokine secretion is shown for all stimulation experiments in Figure 2.

The cytokine pattern reveals differences in the mode of stimulation of the immune system. Overall, RSV-F Fuc- generates a stronger pro-inflammatory and Th2-based response, while RSV-F strongly suppresses the inflammation process and the T-cell response is more Th1-pronounced. Details of the cytokine pattern are discussed below.

Only in the initial phase of the cultivation can a stronger pro- and anti-inflammatory response be observed for RSV-F-stimulated cells. After restimulations at day 7, 14, and 21 the pro- and anti-inflammatory cytokine secretions show individual characteristics, especially differences in the particular duration of their release. In general, the pro-inflammatory response is higher in the afucosylated viral antigene than in the fucosylated one. In particular, TNF-α secretion clearly correlates with the time points of restimulation. In contrast, the anti-inflammatory IL-10 response is more strongly pronounced in RSV-F. As a result, the IFN-γ response is suppressed after the first restimulation on day 7 in the wild-type control. IL-10 shifts the immune response indirectly in favor of Th2 cells, B-cells, and the humoral response, while IFN-γ suppresses the same. In RSV-F Fuc- stimulated cells the Th2 activation is mediated via the immunologically-relevant secretion of IL-4. While its initial secretion is low, the response to restimulations is well-modulated and indicates a highly specific mode of action for the afucosylated RSV-F protein. RSV-F stimulated cells lack this prominent Th2 activating response. Overall, the observed concentrations of Th1 activating IL-2 are unexpectedly low. RSV-F treatment always shows elevated IL-2 levels one day after restimulation. In contrast, the highest IL-2 concentration is reached on day 7 after RSV-F Fuc- treatment and is heavily delayed from the initial stimulation. IL-2 is bound to the IL-2 receptor during T-cell proliferation, which might lead to lower IL-2 concentrations in the cell culture supernatant. In contrast, IFN-γ secretion, which is one of the effector cytokines of activated Th1 cells, is elevated. Hence, activation of Th1 cells can be concluded and a well-balanced Th1/Th2 response is demonstrated. Finally, GM-CSF secretion of RSV-F Fuc- stimulated cells corresponds with each restimulation, but shows decreasing concentrations. GM-CSF promotes the development of monocytes and granulocytes and, thus, facilitates the cellular response and adaptive immunity, since monocytes can mature into dendritic cells.

Unstimulated cells (negative control) exhibit two stress responses. Within the first days of cultivation levels of TNF-α and IL-10 are already increased, which is true for all cytokines, with the exception of TNF-α, from day 16. The initial release of proinflammatory cytokines and chemokines can be induced by overnight resting (e.g., after thawing the cells) and is reported for TNF-α, but not for IFN-γ, by Kutscher et al. [31]. Secondly, the bolus for negative controls contained medium in the same volume as for the vaccine stimulations. Thus, necrotic cells may be whirled up, disintegrated at once, and raise interferon levels of healthy cells nonspecifically. As a result, IL-2 concentration increases at day 21. More importantly, PBMCs react or die spontaneously at this time point due to the absence of any kind of stimulation. This is in line with the normal lifespan of naïve and resting T-cells in vitro, which need to be stimulated with cytokines for survival or proliferation [32,33].

### 3.3. Quality of RNA Preparations and Nanostring Assays

The nCounter immunology panel contains a broad range of genes, covering cytokines, their respective receptors, proteins from signaling pathways, as well as humoral and cellular, or innate and adaptive, immunity-related factors. Thus, a deep and precise insight into the immunologic processes can be gained and modes of action are revealed.

For NanoString experiments 50 to 100 ng of RNA are required. Cells from stimulation experiments in multititer plates yielded RNA in sufficient concentrations (25.8 to 61.7 ng/µL; >20 ng/µL) are required in good quality and purity. In Nanodrop 1000 measurements all samples were within the required ranges for the absorption ratios of 260/280 and 260/230, with the exception of four. However, these samples showed clear peaks for 28S and 18S rRNA and yielded high RNA quality numbers when analyzed with the Fragment Analyzer. Since other samples with good performance in the Nanodrop measurement showed slight fragmentation or low concentrations, we followed the manufacturer’s advice to increase the quantity of RNA used for the NanoString measurement to 150 ng (quantified with Qubit directly before the NanoString sample preparation).

Finally, quality control within the nSolver analysis software showed no flags indicating fragmentation, but consistent binding densities from lane to lane, as well as consistent RNA concentration, indicated by low normalization factors (0.85–1.69). Dilutions of the positive spike in controls showed high linearity within each sample (R^2^ > 0.9875). The reference genes *GAPDH*, *OAZ1*, and *TUBB* were excluded from the panel of used reference genes due to their high coefficient of variation (>75%), as well as the commonly-used reference gene *B2M*, known to be regulated by IFN-γ [34].

### 3.4. Gene Expression Analysis

**Figure 3:**
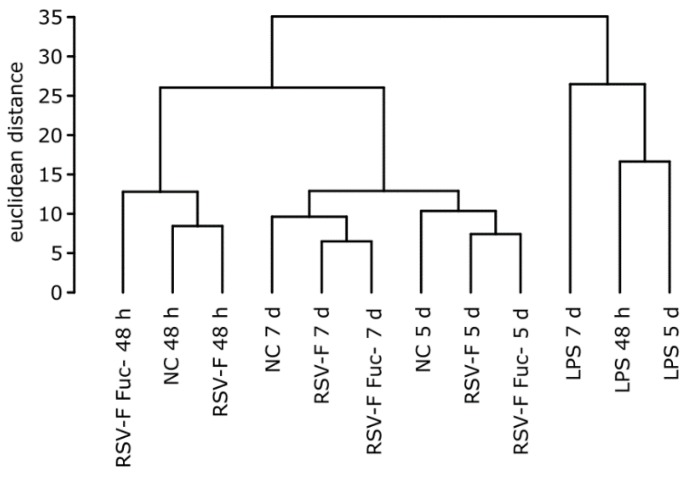
Hierarchical clustering of stimulated samples with normalized and log transformed counts from the NanoString experiment. Samples show time-dependent and stimulus-dependent similarity. Notably, after 48 h the gene expression pattern of cells stimulated with the afucosylated RSV-F protein shows less similarity to the pattern of the wild-type RSV-F-stimulated cells than the latter one to that of the unstimulated negative control (NC).

To get a general idea of changes of the gene expression between stimulants we performed a cluster analysis (Figure 3) using hclust in R (version 3.3.2). As expected, these clusters are mainly time-dependent, with the exception of LPS. Both RSV-F protein-stimulated cells show higher dissimilarity of LPS samples compared to the unstimulated negative control, due to the different mode of action of LPS. Interestingly, after 48 h of cultivation, RSV-F Fuc- is clustered outside the closer-related fucosylated RSV-F and negative control. This indicates that the onset of immunologic reactions is stronger for the afucosylated viral antigene.

**Figure 4:**
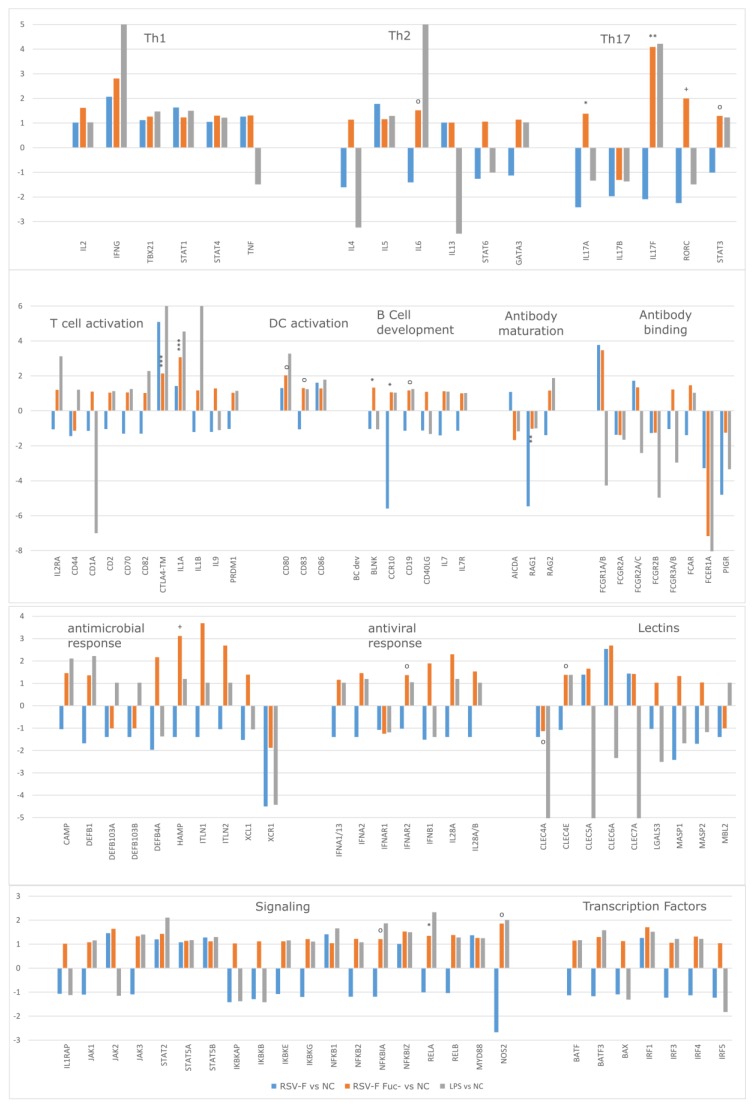
Fold change ratios of stimulated cells in functionally-ordered clusters. Significance is calculated for gene expression of RSV-F Fuc- vs. RSV-F. NanoString nCounter assays were performed in duplicate. Since PBMC are a mixture of cells, higher *p*-values were depicted, as well: *p* ≤ 0.001: ***; *p* ≤ 0.01: **; *p* ≤ 0.05: *; *p* ≤ 0.1: +; and *p* ≤ 0.2: o.

To analyze differences of gene expression pattern of this time point in more detail, fold-change estimates were built from the nCounter assay for each stimulation type. We did not use any specific thresholds to identify genes with a particularly strong fold change, but identified significantly differentially-expressed genes between both RSV-F protein treatments with the help of NanoStringDiff R package. Clusters of genes that are functionally associated with the identified genes or of primary immunologic function were grouped and further analyzed. In accordance with the hclust analysis the early response after 48 h of cell stimulation showed the most prominent and informative differences in gene expression (Figure 4), whereas data of day 5 and 7 often showed transient effects over time (data not shown).

Several clusters show comprehensive upregulation of gene expression for RSV-F Fuc- but not for RSV-F, stimulation after 48 h of stimulation. These clusters cover cellular, humoral, innate, and adaptive immunity, as well as the related transcription factors or activation markers and are discussed in detail.

To augment the cytokine analysis, clusters of Th1-, Th2-, and Th17-related genes were analyzed. Apparently, differences in the expression of Th1-associated genes are negligible, however, IL2 and IFNG (coding for IL-2 and IFN-γ) were upregulated—providing evidence that RSV-F Fuc- evokes a certain Th1 response, although IL-2 concentrations were comparably low in cell culture supernatants of RSV- Fuc- treated cells. In the Th2 panel all interleukins and transcription factors are upregulated in RSV-F Fuc-, but not in RSV-F, which is in compliance to the cytokine data from the HuALN^®^ experiment. The Th17 panel shows the most significant differences in the expression of Interleukin 17 subunits and Th17 associated transcription factors (RORC, STAT3). Activation of Th17 cells is favorable, since they play critical roles in host defense against pathogens at mucosal sites [35], where RSV infections takes place. Both Th2, as well as Th17, promote B cell development and interact closely with B cells in response to pathogens. Th17 cells are involved in B cell recruitment, and Th17 activity may encourage antibody production [36], which is promising since a high titer of neutralizing antibodies is often seen as an indicator for successful RSV protection [37].

B cell development markers show significantly higher expression by the afucosylated viral protein than in wild-type RSV-F. Among them is, e.g., BLNK, which plays a critical role in orchestrating the pro-B cell to pre-B cell transition. Furthermore, genes that code for receptors for the Fc region (*FCGRs* and *FCAR*) of monomeric or complexed immunoglobulins (Ig) are partially activated. They have comprehensive functions in innate, adaptive, and humoral immune responses by modulating subsequent effector and regulatory processes, like phagocytosis of immune complexes and antibody production. The upregulation of these genes proves that immune cells respond to the inflammatory reaction by going into an alert state and expect a humoral immune reaction. The upregulation of IgG and IgA, but not IgE is of interest, since both classes of antibodies transcytose across the epithelial cells to enter the lumen of the respiratory tract, bathes the mucosal lining, and can protect against RSV disease [38,39].

Along with this, there is an overall increased expression of observable genes associated with the activation, differentiation or proliferation of T cells and DCs in RSV-F Fuc-. This is accompanied with a generally stronger activation of parts of the innate immune system comprising antiviral and antimicrobial components, as well as lectins in cells stimulated with RSV-F Fuc-, but not in wild-type RSV-F-stimulated cells. Moreover, the expression of chemotactic ligands and receptors is more pronounced in the afucosylated RSV-F protein. Lastly, RSV-F Fuc- shows a comprehensive upregulation of signaling molecules of the JAK/STAT and NFKB pathway, which leads to broad activation of transcription factors.

LPS, a widely used inflammation control, worked efficiently in this study. It generated a strong pro-inflammatory response and also activated LPS-induced genes like *CD14* (a co-receptor of TLR 4) and other typical LPS-induced chemokines (CCL2, CCL19, and CCL20). However, in comparison to both RSV-F proteins, it failed to activate the Fc receptor expression. In further studies, a more specialized positive control can be used, consisting of a mix of different stimulants which, themselves, address distinct cell types and their pathways. Thus, an encompassing activation of the immune system may be elicited.

Since PBMCs are a mixture of cells, differential gene expression may be masked and a strong activation of a small fraction of cells cannot be distinguished as significant. Thus, we accepted somewhat lower *p*-values within the analysis of differential gene expression. Optimally, cells need to be separated (e.g., into Th1, Th2, Th17, and DC) and analyzed individually to allow an exact assignment of significant changes in the gene expression. A higher number of tests will be necessary; however, smaller individual gene sets will be appropriate in subsequent experiments.

The humoral immune response can be further investigated in the HuALN^®^ system. The experimental setup was not designed for a deeper analysis of B-cells and any generated antibodies. Nevertheless, we see an upregulation of the Recombination Activating Gene 2 (*RAG2*) after 48 h of stimulation for the afucosylated F-protein, but not for the wild-type F-protein. However, the expression of *RAG1* and Activation-Induced Cytidine Deaminase (AICDA), which is heavily involved in somatic hypermutation, is downregulated. This may represent a kind of intermediate incomplete activation status due to the lack of additional factors (e.g., stromal cells). Recently, the physiology of the HuALN^®^ system has been further improved by establishing a differentiation protocol to generate lymph node stromal-like cells from mesenchymal stromal cells for co-cultivation in the existing allogenic PBMC and mDC system [40]. We believe that this improvement can further enhance the analysis of relevant glycoengineered viral antigenes.

Investigation of the glycosylation pattern of the produced antibodies or other immunologically-important molecules is highly interesting in itself, since it correlates with the vaccine’s mode of action. Mahan et al. revealed that distinct glycosylation patterns are programmed, and even remembered, after immunization, when adjuvant stimulation addressed Toll-like receptors (TLRs) [41]. However, the responsible glucuronyltransferases are only partially covered by the used NanoString panel, and have to be analyzed with custom assays in the future.

The analysis of cytokines and of the gene expression within suggests different modes of activation between both RSV-F types, which can be attributed to the fucosylation state of the RSV-F protein. Afucosylation led to a much stronger pro-inflammatory Th2 and Th17 based activation. However, in this study we were unable to address the molecular mechanism of this effects experimentally. It is known, that the interaction of immune receptors could be mediated by the degree of fucosylation of ligand glycans. The most prominent example is the improved binding of fucose-deficient IgG1 to human FcγRIIIA [42]. Furthermore, fucosylation of glycans may influence the conformation of the RSV-F proteins. In this context, unmasking of complete epitopes is unlikely due to the small size of fucose, but increasing accessibility of epitopes by the immune system might be possible. A second explanation for the observed small, but significant, differences in immunogenic potency of RSV-F and RSV-F Fuc- might be the proportion of remaining pep27 at the purified proteins. Although we cannot detect significant differences by semiquantitative densitometry, an influence of pep27 on immunogenicity is suggested. An immunomodulatory role has been found for bovine RSV pep27 [43]; however, no such role has been attributed to the human RSV intervening peptide, which does not share a sequence similarity with its bovine counterpart.

However, most importantly, in this study we showed that the HuALN^®^ reactor and the downstream analysis methods are able to discriminate the effects caused by very small modifications of the glycan structure.

Recently, Rosenlöcher et al. reported the gradual fucosylation of recombinant glycoproteins by exploiting the salvage pathway of the same cells used in this study [24]. Supplementation of the culture media with distinct concentrations of fucose could allow a fine tuning of the Th1/Th2 balance and the strength of the inflammatory process. Furthermore, by supplementing immunogenic fucose analogues in the culture media, the immunogenicity of recombinant protein vaccines could be further modulated.

## 4. Conclusions

The complementary quantification of cytokines in the cell culture supernatant and analysis of the gene expression by extensive nCounter panels provided the possibility to analyze the extent of the activation of the immune system broadly. Altogether, we proved with the chosen methods that viral proteins with varying glycan structures are able to trigger the immune system in a slightly different way. Afucosylated RSV-F reveals stronger activation of the innate immune system, generates a moderate inflammation response, and activates, via Th2 and Th17, the humoral immune system.

We suggest that through gradual afucosylation of the RSV-F protein N-glycans a balance between Th1 and Th2 response might even be more controllable. Further characterization of the adjuvant and the RSV vaccine has to concentrate on the analysis of the produced antibodies.
